# Opioid Prescribing Patterns of Board-Certified Emergency Physicians Compared With Other Physicians Practicing Emergency Medicine Among Medicare Part-D Beneficiaries Between 2018 and 2020 in the United States

**DOI:** 10.1016/j.acepjo.2025.100324

**Published:** 2026-01-19

**Authors:** Yachana Bhakta, Michael Gottlieb, Susan E. Farrell, Suzanne R. White, Kevin B. Joldersma, Melissa A. Barton, Chadd K. Kraus, Lyndsay Tyler, Bradley Chappell, Jonah Geddes, Diane L. Gorgas

**Affiliations:** 1 American Board of Emergency Medicine, East Lansing, Michigan, USA; 2 Department of Emergency Medicine, Rush University Medical Center, Chicago, Illinois, USA; 3 Department of Emergency Medicine, Brigham and Women’s Hospital, Boston, Massachusetts, USA; 4 Department of Emergency Medicine, Wayne State University, Detroit, Michigan, USA; 5 Department of Emergency and Hospital Medicine, Lehigh Valley Health Network, Allentown, Pennsylvania, USA; 6 American Osteopathic Board of Emergency Medicine, Chicago, Illinois, USA; 7 Oregon Health & Science University - Portland State University School of Public Health, Portland, Oregon, USA; 8 Department of Emergency Medicine, Wexner Medical Center, The Ohio State University, Columbus, Ohio, USA

**Keywords:** *board certification*, *emergency medicine*, *opioid*, *opioid prescription*, *pain management*, *emergency department*

## Abstract

**Objectives:**

There is limited knowledge regarding whether board-certified emergency medicine (EM) physicians have different opioid prescribing rates compared to other physicians working in emergency departments (EDs). This study aims to determine if opioid prescribing rates differ based on board-certification status compared with other physicians practicing in an ED setting.

**Methods:**

An IRB-approved, cross-sectional analysis was performed on the Medicare Part D Prescribers by Provider and Drug datasets from 2018 to 2020 to determine prescribing rates of EM physicians by board-certification status with the American Board of Emergency Medicine or the American Osteopathic Board of Emergency Medicine. EM physicians prescribing opioids to 11 or more Medicare beneficiaries per calendar year in EDs were included. The average total day supply (TDS) of opioids per beneficiary for the 4 most common opioids (acetaminophen/codeine, hydrocodone/acetaminophen, oxycodone/acetaminophen, tramadol) was estimated using generalized linear models with Poisson distribution, log-link, and individual clustering and compared by board certification in EM status. Incident rate ratios (IRR) were used to describe the association between board certification in EM and the average TDS of opioids.

**Results:**

A total of 29,144 physicians were included in the study, with 23,720 (81.4%) board-certified by the American Board of Emergency Medicine or the American Osteopathic Board of Emergency Medicine and 5424 (18.6%) certified by another specialty board (eg, family medicine [48.6%], internal medicine [17.3%]). Physicians working in EDs, who were not board-certified in EM, prescribed a higher average TDS compared with board-certified EM physicians: acetaminophen/codeine (TDS, 10.8 vs 4.0; IRR, 2.7 [95% CI, 2.3-3.2]); hydrocodone/acetaminophen (TDS, 15.2 vs 3.8; IRR, 4.0 [95% CI, 3.7-4.4]); oxycodone/acetaminophen (TDS, 19.8 vs 4.4; IRR, 4.5 [95% CI, 3.8-5.3]); tramadol (TDS, 20.1 vs 5.3; IRR, 3.8 [95% CI, 3.5-4.1]).

**Conclusions:**

Board certification in EM was associated with lower opioid prescribing rates. The average TDS of opioids per Medicare beneficiary was lower for board-certified EM physicians compared to non-EM board-certified physicians staffing EDs between 2018 and 2020.

## 1 Introduction

### 1.1 Background

Regulatory policies and clinical practice guidelines have focused on treating pain but have a goal of appropriate reductions in opioid prescribing in response to the opioid epidemic.^1^^,^^2^ Opioid-related overdose deaths nearly quadrupled from 21,089 in 2010 to 81,806 in 2022, with approximately 15,000 of those deaths linked to opioid prescriptions in 2022.^3^ The rising mortality from opioid overdoses and the Centers for Disease Control and Prevention’s (CDC) review of the effectiveness and risks of opioid use prompted the CDC to publish opioid prescribing guidelines.^2^^,^^4^

Prior research indicates an overall decreasing trend in opioid prescribing in connection with the publication of the CDC Guidelines on Prescribing Opioids for Chronic Pain, along with other state policies and clinical guidelines.^4^, ^5^, ^6^ These policies and guidelines aim to minimize the harms associated with opioid use by improving communication with patients regarding the risks of opioid use and by enhancing patient care safety through adjustments in prescribing alternative analgesics.^4^, ^5^, ^6^ According to the CDC, the number of opioid prescriptions dispensed has decreased significantly from 2019 to 2023, with the opioid dispensing rate falling from 46.8 to 37.5 per 100 persons.^7^ Despite this decline in prescribing rates, prior research suggests considerable inter- and intra-physician variability in opioid prescribing practices across medical specialties and healthcare settings.^8^, ^9^, ^10^

### 1.2 Importance

Patients presenting to emergency departments (EDs) with acute pain are often prescribed a short course of an opioid for pain management.^9^^,^^11^, ^12^, ^13^ Although episodic prescribing during ED visits has been shown to contribute only minimally to the overall supply of prescribed opioids,^14^^,^^15^ emergency medicine (EM) physicians and EDs continue to play a crucial role in ensuring appropriate opioid prescribing and in caring for patients experiencing adverse effects from opioids or opioid use disorder.^16^

Previous studies have demonstrated differences in opioid prescribing rates between physicians and nonphysicians in the ED.^17^ However, limited knowledge exists regarding whether opioid prescribing patterns vary based on the board-certification status of physicians working in the ED.

### 1.3 Goals of this Investigation

This study aims to examine whether there are differences in opioid prescribing rates between American Board of Emergency Medicine (ABEM)- and American Osteopathic Board of Emergency Medicine (AOBEM)-certified physicians and those who are not ABEM- nor AOBEM-certified but are practicing in the ED.

## 2 Methods

### 2.1 Study Design

We conducted a cross-sectional study using the Medicare Part D Prescribers by Provider and Drug datasets from 2018 to 2020 along with physician-level data from the ABEM, the AOBEM, and the American Board of Medical Specialties (ABMS) to examine prescribing rates by board-certification status. This study was deemed exempt by the WIRB-Copernicus Group Institutional Review Board. This study follows the Strengthening the Reporting of Observational Studies in Epidemiology reporting guidelines for cross-sectional studies.^18^

### 2.2 Data Sources

The Medicare Part D Prescribers by Provider and Drug datasets include healthcare professionals’ prescribing information to Medicare beneficiaries with a Part D prescription drug plan. The datasets are organized by National Provider Identifier (NPI) and include information on the specific drugs prescribed, the total number of prescriptions, the total daily supply (TDS) dispensed, drug costs, and geographical location. Data were available for all providers with ≥11 claims for a given drug in the data set.^19^ Administrative datasets from ABEM, AOBEM, and the ABMS included physician demographics and years of active certification. All datasets were linked using NPI numbers and surnames.

### 2.3 Selection of Participants

All opioid prescription claims for Medicare Part D beneficiaries and prescribers with the specialty code of “Emergency Medicine” between January 1, 2018, and December 31, 2020, were identified. The primary taxonomy provided by the National Plan and Provider Enumeration System National Provider Registry was used to distinguish physicians from other prescribers, such as nurse practitioners and physician assistants. Physicians were included in the study if they prescribed 1 of the 4 most common opioids within the dataset to ≥11 beneficiaries per year between 2018 and 2020: acetaminophen/codeine, hydrocodone/acetaminophen, oxycodone/acetaminophen, or tramadol. Only physicians prescribing opioids in the 50 United States (US) and the District of Columbia were included in the study. Aggregate records derived from providers with fewer than 11 claims for a specific drug are excluded from the Part D Prescribers by Provider and Drug datasets.^19^ Additionally, the Centers for Medicare & Medicaid Services suppresses the number of total beneficiaries for providers with claims for less than 11 beneficiaries;^19^ therefore, providers with claims for less than 11 beneficiaries were excluded from the dataset. Data on specialty and ABMS board-certification status between 2018 and 2020 were determined through cross-matching physician data from the study Medicare Part D dataset with data registries maintained by ABEM, AOBEM, and the ABMS. Physicians were categorized as board-certified if they held an ABEM or AOBEM certification in EM at any point during 2018 and 2020.

### 2.4 Measurements

Physician characteristics such as sex, age group, medical degree, years in practice, specialty, number of beneficiaries who were provided prescriptions, and geographic prescribing region were collected. The age of each physician during the prescribing year was determined by calculating the time between a physician’s birth year and the prescribing year. The physicians were organized into age groups based on their average age during the study period. The number of years in practice for each physician was determined by calculating the number of years between graduation from medical school and the prescribing year. Physicians were categorized into groups based on the average number of years in practice during the study period. Geographic prescribing region was reported based on the US Census Bureau’s categorization of states into geographic regions of the US.^20^

### 2.5 Exposures

The exposure of interest for this study was board certification in EM. Physicians were categorized as board-certified if they held an active board certification in EM with either ABEM or AOBEM between January 1, 2018, and December 31, 2020. The board-certification status was determined using administrative datasets managed by ABEM and AOBEM, respectively.

### 2.6 Outcomes

The outcomes of average TDS per beneficiary of the four most prescribed opioids in the dataset—acetaminophen/codeine, hydrocodone/acetaminophen, oxycodone/acetaminophen, and tramadol—between 2018 and 2020 were examined. The average TDS per beneficiary for each physician by opioid was generated by dividing the aggregate TDS prescribed by the total number of beneficiaries with at least 1 claim for the opioid being examined. The average TDS per beneficiary for each opioid was evaluated by the physician’s board-certification status in EM.

### 2.7 Data Analysis

Descriptive statistics were used to summarize physician characteristics. A generalized linear model using Poisson, log-link, and individual clustering for each of the 4 most prescribed opioids in the study was built. Average TDS per beneficiary was modeled with board-certification status as a primary predictor and year as a covariate to adjust for temporal trends. The models were adjusted for the effects of the calendar year due to the observed decreases in the number of opioid claims over time.^21^^,^^22^ Standard errors were clustered at the physician level to account for within-provider correlation. Model assumptions and fit were assessed using the Goodness-of-Fit test and examining Pearson and deviance residuals. Among Medicare-eligible physicians who practice in EM, the models were used to determine the association between a physician’s board-certification status and the average TDS of opioids prescribed per beneficiary. The strength of the association was measured through the adjusted incident rate ratios (IRR). Statistical significance was assessed using an alpha of 0.05. The average TDS of opioids per Medicare beneficiary was compared between board-certified physicians and physicians with an NPI taxonomy of EM who were not ABEM- nor AOBEM-certified. Maximum likelihood estimation was used to estimate model coefficients, and 95% Wald confidence intervals (CIs) were calculated using cluster-robust standard errors. All model outputs were reported as average TDS of opioids, average absolute differences in TDS, and IRRs with 95% CIs. All statistical analyses were conducted using Stata Version 19 (StataCorp).

## 3 Results

A total of 29,144 physicians with a taxonomy of EM were included for analysis (Figure). Among those, 23,720 (81.4%) were board-certified and 5424 (18.6%) listed EM but were not board-certified in EM. Overall, the number of physicians prescribing opioids in this study decreased from 24,266 physicians in 2018 to 18,967 physicians in 2020. From 2018 to 2020, the number of board-certified physicians prescribing opioids decreased by 21.6% (19,836 to 15,546 physicians) and the number of other physicians decreased by 22.7% (4430 to 3421 physicians).

**Figure 1:**
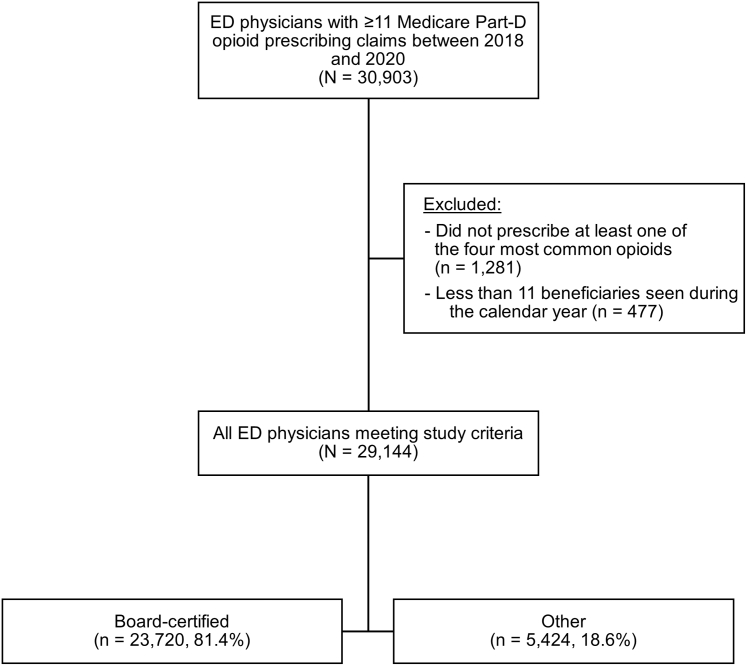
Study population flow diagram. Abbreviations: ED, emergency department.

Both the board-certified and nonboard-certified physicians were mostly male (69.0% vs 66.5%, respectively) and more likely to be graduates of allopathic medical schools (78.4% vs 63.0%, respectively). Most board-certified emergency physicians had up to 19 years after graduation (75.3%) compared with 20 years or more of experience after graduation for other physicians (52.7%) in the study. When compared with board-certified physicians, nonboard-certified physicians were most likely to be older (53.0 years vs 44.8 years) and practice in the South (52.6% vs 37.3%, respectively). Almost half of the other physicians were certified in family medicine (48.6%), followed by internal medicine (17.3%), pediatrics (0.9%), and surgery (1.5%). The specialty data for 1650 (30.4%) of other physicians were missing (Table 1).

**Table 1:** Baseline characteristics of physicians prescribing one of the four most common opioids to Medicare Part-D beneficiaries in the emergency department between 2018 and 2020 by emergency medicine board-certification status, *n* = 29,144.

Characteristics	Board-certified (*N* = 23,720)	Other (*N* = 5424)
**No. (%) with data**	81.4%	18.6%
**Year**		
2018	19,836 (83.6%)	4430 (81.7%)
2019	18,653 (78.6%)	4038 (74.4%)
2020	15,546 (65.5%)	3421 (63.1%)
**Sex**		
Female	5886 (24.8%)	653 (12.0%)
Male	16,369 (69.0%)	3607 (66.5%)
Missing	1465 (6.2%)	1164 (21.5%)
**Age**^a^	44.8 (10.3)	53.0 (10.7)
**Age group**^b^		
20-29	181 (0.8%)	41 (0.8%)
30-39	8665 (36.5%)	573 (10.6%)
40-49	7790 (32.8%)	879 (16.2%)
50-59	4497 (19.0%)	1444 (26.6%)
60+	2577 (10.9%)	1338 (24.7%)
Missing	10 (<0.1%)	1149 (21.2%)
**Medical degree**		
DO	5082 (21.4%)	720 (13.3%)
MD	18,588 (78.4%)	3417 (63.0%)
MBBS or MBBCh	45 (0.2%)	48 (0.9%)
Missing	5 (<0.1%)	1239 (22.8%)
**Years of practice**^c^		
<10	10,273 (43.3%)	617 (11.4%)
10-19	7590 (32.0%)	777 (14.3%)
20-29	3736 (15.8%)	1278 (23.6%)
30+	996 (4.2%)	1578 (29.1%)
Missing	1125 (4.7%)	1174 (21.6%)
**Specialty**		
Emergency medicine	23,720 (100.0%)	(0.0%)
Family medicine	0 (0.0%)	2636 (48.6%)
Internal medicine	0 (0.0%)	936 (17.3%)
Surgery	0 (0.0%)	81 (1.5%)
Pediatrics	0 (0.0%)	51 (0.9%)
Other	0 (0.0%)	70 (1.3%)
Missing	0 (0.0%)	1650 (30.4%)
**Number of beneficiaries by year**		
2018	725,882	223,246
2019	646,045	188,356
2020	452,363	138,592
**Number of +65 beneficiaries by year**		
2018	175,229	74,408
2019	143,078	58,749
2020	86,523	39,300
**Region**^d^		
Midwest	6185 (26.1%)	1393 (25.7%)
Northeast	3120 (13.2%)	511 (9.4%)
South	8841 (37.3%)	2855 (52.6%)
West	5832 (24.6%)	691 (12.7%)

a The average physician age for the study period was calculated and is reported with the standard deviation.

b The average physician age during the time period was calculated and is reported by age group.

c The average number of practice years during the time period was calculated and is reported by group of practice years.

d Percentages do not add up to 100.0%. Physicians prescribing region varied between 2018 - 2020.

The overall average TDS of opioids prescribed by nonboard-certified emergency physicians was significantly higher than board-certified emergency physicians for all opioids in the study: acetaminophen/codeine (TDS, 10.8 vs 4.0; MD, 6.8 [95% CI, 5.2-8.4]; IRR, 2.7 [95% CI, 2.3-3.2]); hydrocodone/acetaminophen (TDS, 15.2 vs 3.8; MD, 11.4 [95% CI, 10.3-12.6]; IRR, 4.0 [95% CI, 3.7-4.4]); oxycodone/acetaminophen (TDS, 19.8 vs 4.4; MD, 15.4 [95% CI, 12.6-18.3]; IRR, 4.5 [95% CI, 3.8-5.3]); tramadol (TDS, 20.1 vs 5.3; MD, 14.8 [95% CI, 13.3-16.3]; IRR, 3.8 [95% CI, 3.5-4.1]) (Table 2).

**Table 2:** The average total day supply of opioids prescribed to Medicare Part-D beneficiaries between 2018 and 2020 by emergency medicine board-certification status.

Opioid	Year	TDS among board-certified physicians *Mean (95% CI)*	TDS among nonboard-certified physicians *Mean (95% CI)*	Absolute difference *Mean (95% CI)*	Incident rate ratios (95% CI)
Acetaminophen/codeine	Overall	4.0 (3.8-4.2)	10.8 (9.2-12.4)	6.8 (5.2-8.4)	2.7 (2.3-3.2)
	2018	4.0 (3.7-4.2)	10.7 (9.2-12.3)	6.8 (5.2-8.3)	2.6 (2.2-3.0)
	2019	3.9 (3.7-4.2)	10.6 (9.0-12.3)	6.7 (5.1-8.3)	2.8 (2.3-3.3)
	2020	4.2 (3.8-4.5)	11.2 (9.3-13.1)	7.1 (5.3-8.9)	2.8 (2.3-3.5)
Hydrocodone/Acetaminophen	Overall	3.8 (3.7-3.9)	15.2 (14.0-16.4)	11.4 (10.3-12.6)	4.0 (3.7-4.4)
	2018	3.9 (3.7-4.0)	15.5 (14.4-16.7)	11.7 (10.5-12.9)	4.1 (3.7-4.4)
	2019	3.7 (3.6-3.9)	15.0 (13.7-16.2)	11.2 (10.0-12.4)	4.0 (3.6-4.4)
	2020	3.8 (3.6-3.9)	15.2 (13.9-16.4)	11.4 (10.2-12.6)	4.0 (3.6-4.4)
Oxycodone/Acetaminophen	Overall	4.4 (4.0-4.7)	19.8 (16.9-22.7)	15.4 (12.6-18.3)	4.5 (3.8-5.3)
	2018	4.2 (3.9-4.6)	19.1 (16.5-21.7)	14.9 (12.2-17.5)	4.3 (3.6-5.0)
	2019	4.4 (4.0-4.7)	19.7 (16.7-22.7)	15.4 (12.4-18.3)	4.6 (3.8-5.5)
	2020	4.7 (4.2-5.2)	21.2 (17.6-24.7)	16.5 (13.1-19.9)	4.9 (4.0-6.1)
Tramadol	Overall	5.3 (5.0-5.5)	20.1 (18.6-21.5)	14.8 (13.3-16.3)	3.8 (3.5-4.1)
	2018	5.3 (5.0-5.6)	20.1 (18.7-21.5)	14.8 (13.3-16.2)	3.8 (3.5-4.1)
	2019	5.0 (4.7-5.2)	18.9 (17.4-20.3)	13.9 (12.5-15.3)	3.7 (3.3-4.0)
	2020	5.8 (5.4-6.2)	22.0 (20.1-23.9)	16.2 (14.4-18.0)	4.0 (3.5-4.4)

Note: IRRs are reported and use the average TDS prescribed by board-certified in EM as the reference. Models were adjusted for the effects of the calendar year.

Abbreviations: IRR, incident rate ratio; CI, confidence interval; TDS, total day supply; EM, emergency medicine.

## 4 Limitations

This study analyzed physicians with NPI taxonomies identified as EM physicians who wrote prescriptions for Medicare beneficiaries with Part D coverage. Other physicians with or without board certification in EM may have been missed in this database (eg, physicians practicing in the Veterans Administration health system, physicians who only prescribed opioids to non-Medicare beneficiaries in a given year). Additionally, prescribing rates in this study may also miss those who opted to pay for the prescriptions out-of-pocket. Another limitation of this study is the use of the Medicare provider specialty code, based on the NPI taxonomies. Physicians who practice in multiple specialties or multiple healthcare settings in addition to the ED were not included in the study if more than half of a physician’s claims were filed for another specialty or setting. Additionally, this study does not consider the potential confounding impact of age or years of practice on opioid prescribing rates.^8^ Furthermore, we were not able to account for potential confounding by practice setting (eg, urban vs rural setting) or geographic region. It is possible that nonboarded physicians may practice in geographic regions where a larger proportion of patients need opioid prescriptions for conditions related to cancer, chronic pain management, or acute injuries. These patients may seek care in the ED if access to primary care is more limited in a given area. Although board certification in EM was associated with lower opioid prescribing, we were not able to demonstrate causality. Finally, opioid prescribing rates are not patient-centric outcomes. Although we report rates of overall opioid prescribing, we cannot comment on the appropriateness of the prescription indication. Therefore, it also remains possible that board-certified physicians may be underprescribing opioids in select cases.

## 5 Discussion

Among the 29,144 physicians included in the study between 2018 and 2020, there was a markedly higher rate of opioid prescribing among nonboard-certified physicians compared with those who were board-certified. This finding remained consistent across all 4 of the most commonly prescribed opioids, highlighting an important difference between groups that is particularly salient within the current opioid epidemic. Although opioid prescribing rates in EDs have dropped over time, continued opioid stewardship while ensuring good pain control is necessary to help curb the opioid epidemic. A better understanding of the mechanisms driving differences in prescribing rates between board-certified EM and nonboard-certified physicians practicing in the ED may help inform practices or strategies for safer opioid prescribing and effective pain management for patients in the ED.

Previous studies have found that the average number of opioid prescriptions per prescriber is higher among family medicine and internal medicine physicians compared with emergency physicians, regardless of practice location.^11^^,^^22^^,^^23^ A variety of factors related to training, continuing education, and department or institutional culture may contribute to the observed differences in TDS between board-certified emergency physicians and non–EM–board-certified physicians working in the ED setting. The curricula for EM residency training programs have a specific and emphasized focus on opioid prescribing in the ED setting in contrast to other specialty residency training programs (eg, family medicine, internal medicine, surgery).^24^ For example, most EM board-certified physicians completed a residency that included topics in opioid use disorder and addiction medicine.^24^ This is further supported by a retrospective chart review conducted that found EM residents prescribed fewer opioids compared with non-EM residents in the ED setting.^25^ As a result, it is likely that EM residency training may contribute in part to these differences in prescribing patterns.

Continuing medical education (CME) for EM board-certified physicians is also tailored for prescribing in the ED setting. Although state-mandated CME requirements are similar for all physicians, EM board-certified physicians may be expected to complete additional CME to meet board-certification requirements, which have a more specialized and specific focus on opioid prescribing in the ED setting. Another factor is that those without EM board certification tended to be older and may not have had the training opportunities focused on opioid prescribing in the ED during residency. They may also have trained during a time when opioid use disorder had garnered less national attention. Moreover, this may also reflect differences in practice mix, with board-certified physicians more commonly working in urban settings.^26^

An additional consideration is the rise of newer strategies such as alternatives to opioids (ALTO) and other campaigns that are emphasized by EM residency training programs and additional strategies for physicians specializing in EM and physicians certified in EM. The adoption of ALTO strategies by board-certified EM physicians may have contributed to an increased use of alternative pain control strategies, thereby reducing opioid prescribing rates over time. ALTO strategies to treat pain in the ED include evidence-based treatment practices such as nonopioid medications, trigger point injections, nitrous oxide, and ultrasound-guided nerve blocks for pain management.^27^ There is evidence of decreased utilization of opioids in EDs after ALTO program strategies were implemented.^27^, ^28^, ^29^ In these instances, it might reflect an issue with knowledge dissemination, where training opportunities regarding opioid-sparing programs are focused on emergency physicians, rather than all physicians working in emergency departments, highlighting an opportunity to further expand programs such as this to all learners.

Tailoring education and training strategies within residency programs, certification processes, and department cultures to increase the use of alternative pain management strategies in the ED and limiting opioid prescribing doses and durations to effectively manage patient pain may positively contribute to improved patient safety and lessen the public health burdens associated with the opioid epidemic. Continued stewardship in opioid prescribing in residency programs, certification processes, and CME is critical in preventing new opioid use disorders and opioid-related deaths. Continued opioid stewardship may focus on safer opioid prescribing practices, alternatives to opioids for pain management, increasing the distribution of naloxone and initiation of buprenorphine for opioid use disorder, and increased use of prescription drug monitoring programs and improvements to patient education. Future analysis of factors that result in differences in opioid prescribing by board certification might inform educational and assessment strategies that improve opioid prescribing patterns for all physicians working in EDs. For example, future research could examine the impact of various continuing education opportunities, such as differential uptake of ABEM’s Opioid Use and Substance Use Disorder modules, on learning and subsequent opioid prescribing rates.

The findings of the present study show that board-certified EM physicians prescribed a significantly lower average TDS of opioids to Medicare beneficiaries compared to other physicians working in EDs from 2018 to 2020. A better understanding of the driving factors of these differences could inform training and education strategies for all physicians staffing EDs to ensure safer opioid prescribing practices and increase the use of alternative pain management strategies to improve patient safety.

## Author Contributions

Study concept and design: YB, MG, CKK, JG

Acquisition, analysis, and interpretation of data: YB, MG, KBJ, CKK, BC, LT, JG

Drafting the manuscript: YB, MAB, MG, DLG, SEF, SRW

Critical revision of the manuscript for important intellectual content: MG, MAB, DLG, SEF, SRW

## Funding and Support

By *JACEP Open* policy, all authors are required to disclose any and all commercial, financial, and other relationships in any way related to the subject of this article as per ICMJE conflict of interest guidelines (see www.icmje.org). The authors have stated that no such relationships exist.

## Conflict of Interest

Dr Barton, Dr Joldersma, Dr Gottlieb, Ms Bhakta, and Ms Tyler are employees of the American Board of Emergency Medicine (ABEM). Dr Gorgas, Dr Farrell, and Dr White are members of the ABEM Board of Directors. ABEM receives revenue for administering the Qualifying Examination and the Oral Certifying Examination. Dr Chappell is the chair of the American Osteopathic Board of Emergency Medicine (AOBEM). AOBEM receives revenue from the written and oral certification exams. Dr Kraus is an editor for JACEP Open.
